# Exploring agreement and feasibility between virtual home visits and in-person home visits for peritoneal dialysis patients—a paired study

**DOI:** 10.1080/0886022X.2022.2049305

**Published:** 2022-03-13

**Authors:** Jin Chen, Bo Zheng, Lijuan Yin, Qin Zhou, Wenshu Liu, Pengli Li, Xiuxiu Zhao, Xiuling Chen, Yi Li, Hanlu Ding, Guisen Li

**Affiliations:** a Renal Department and Institute of Nephrology, Sichuan Academy of Medical Sciences and Sichuan Provincial People’s Hospital, Sichuan Clinic Research Center for Kidney Diseases, School of Medicine, University of Electronic Science and Technology of China, Chengdu, China; b Department of Sanitary Technology, West China School of Public Health and West China Fourth Hospital, Sichuan University, Chengdu, China

**Keywords:** Peritoneal dialysis, telehealth, virtual home visits, agreement, feasibility

## Abstract

**Introduction:**

Virtual home visits may improve chronic disease management. However, whether they are suitable for peritoneal dialysis (PD) patients has not yet been fully investigated. This study aimed to compare the agreement and acceptance of virtual home visits and in-person home visits in PD patients.

**Methods:**

This was a paired, single center, noninferiority trial. Participants received a virtual home visit and an in-person home visit simultaneously. A home visit checklist was built for standardization visits. The content was divided into three parts: domestic habits (57 items), bag exchange procedures (56 items), and exit site care (53 items). Satisfaction questionnaires for both patients and nurses were designed to assess attitudes toward home visits and socioeconomic effects.

**Results:**

A total of 30 PD patients were enrolled in a single center. The information collected from virtual home visits and in-person home visits was found to be highly consistent. The perfect agreement was found in 52/57, 49/56, and 44/53 items (Cohen's kappa 0.81–1.00), substantial agreement in 4/57, 7/56, and 8/53 items (Cohen's kappa 0.61–0.80). Patients reported almost identical satisfaction for virtual home visits and in-person home visits (*Z* = 0.39, *p* = 0.70). PD nurses reported similar feasibility and patient cooperation for the two visit types (*Z* = 0.99, *p* = 0.33; *Z* = 1.65, *p* = 0.10, respectively). In addition, virtual home visits were found to be more cost-effective than in-person home visits.

**Conclusions:**

Virtual home visits information collection was similar to in-person home visits in PD. There were no differences in participant satisfaction and feasibility between the two visit types.

## Background

Peritoneal dialysis (PD) is widely used for end-stage renal disease treatment [1]. PD was introduced in China in the 1960s and has developed rapidly. A 2016 Chinese survey reported an age-adjusted prevalence of PD of 34.99 per million people [2]. The total number of PD patients had increased to 103,348 in 2019. Development of therapeutic technique and patient management improved clinical outcomes in past years [3]. However, regional variation in patient survival was observed in PD centers owing to a lack of standard care protocols and operating procedures [4].

Home visits are an important part of PD patient management that can improve patient survival and reduce peritonitis and hospitalization rates [5–7]. At present, there are no published guidelines for PD home visits in China. It is generally accepted that home visits should evaluate the dialysis environment, PD fluid storage, personal hygiene, and PD skills and knowledge [8]. However, service delivery is not always satisfactory. In a survey of PD home visits in the US, 52% of centers were found to make initial home visits and at least one follow-up visit, 16% made home visits as needed, and 21% did not carry out home visits at all. Home visits represent a major expenditure of time and manpower [9]. During the COVID-19 pandemic, the need for social distancing and self-quarantine has made home visits even more challenging.

Telemedicine has grown rapidly in recent years, and especially during the pandemic. Research in China found 93.8% of Chinese tertiary hospitals provide a wide range of telemedicine services for patients [10], and hypertension and diabetes mellitus are successfully managed *via* telemedicine [11,12]. Online consulting, tele-education and virtual visits are common telehealth services. A virtual visit is the use of internet video technology to transfer information between medical staff and patients and provide a complete medical service [13]. This form of the visit has several benefits, including flexible visiting times, cost and time savings, and high patient satisfaction [14,15]. Virtual visits have proved to be effective tools in the management of chronic disease [16–20]. However, the purpose of virtual visits inpatient management varies and medical protocols need to be designed, implemented and evaluated according to the characteristics of each disease [14,21–24].

Despite eHealth interventions to support PD patients in the delivery and management of home care, evidence of effectiveness is limited [25,26]. In particular, unlike other chronic diseases, PD home visits cannot be fully evaluated by outcome indicators. PD nurses must be familiar with the patient’s daily routines and ensure that patient carefully follows the step-by-step dialysis exchange and exit site care procedure. To the best of our knowledge, no research has been conducted on the effectiveness of PD virtual home visits. Therefore, this study focuses specifically on the consistency of information collection and visit satisfaction between virtual home visits and in-person home visits.

## Methods

### Study design

This study was a paired, single center, noninferiority trial to identify the consistency of information collection and participant satisfaction during virtual home visits and in-person home visits. This study had three phases: checklist and questionnaire design, virtual home visit pilot, and visit implementation. Patients received a virtual home visit and an in-person home visit at the same time. Recruitment occurred from November 1 to 30, 2020. Visits were completed between November 5 and December 8, 2020. The study protocol and consent forms were approved by Sichuan Provincial People’s Hospital (NO. 2020312). All participants provided written informed consent.

### Participants

All participants were recruited from the PD center of Sichuan Provincial People’s Hospital, one of the largest PD centers in Southwest China, and included patients from across the province. Inclusion criteria included age >18 years, on PD >12 months and with previous experience of in-person home visits. Exclusion criteria included the inability of patients or caregivers to complete PD procedures independently, lack of an internet-connected device with a webcam, residence outside central Chengdu city owing to traffic restrictions during the COVID-19 pandemic. One hundred and twenty -three patients were assessed for eligibility, 31 were enrolled. One patient withdrew by breaching the visiting appointment, and 30 completed their visits. Two nephrologists acted as investigators, and four PD nurses carried out the visits.

### Checklist and questionnaire preparation

**Figure 2.**
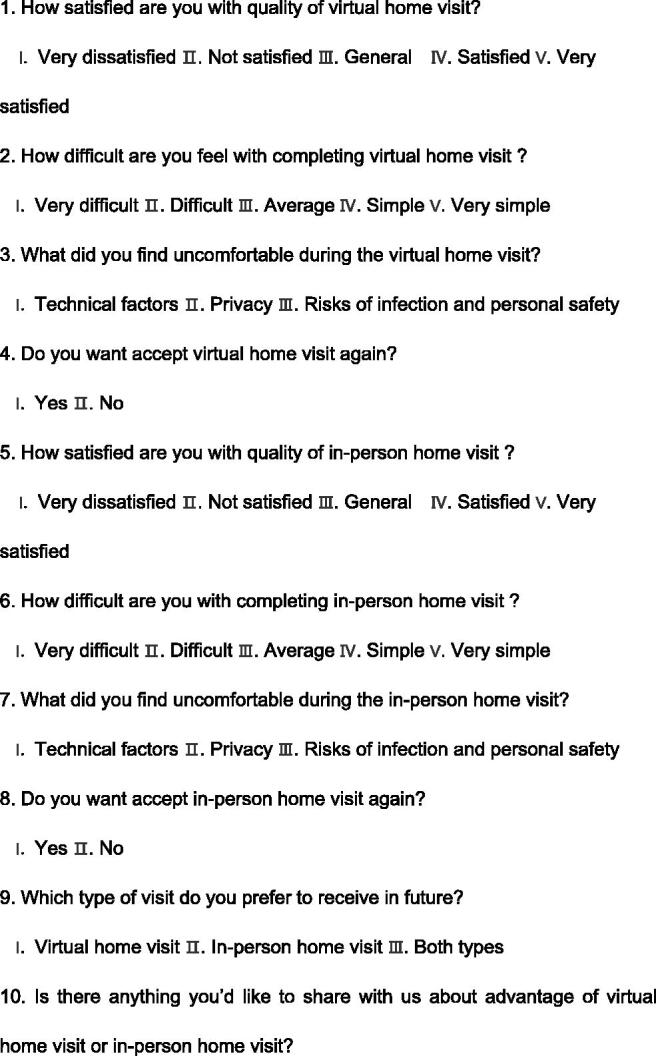
Questionnaire to measure the satisfaction of the patient.

**Figure 3.**
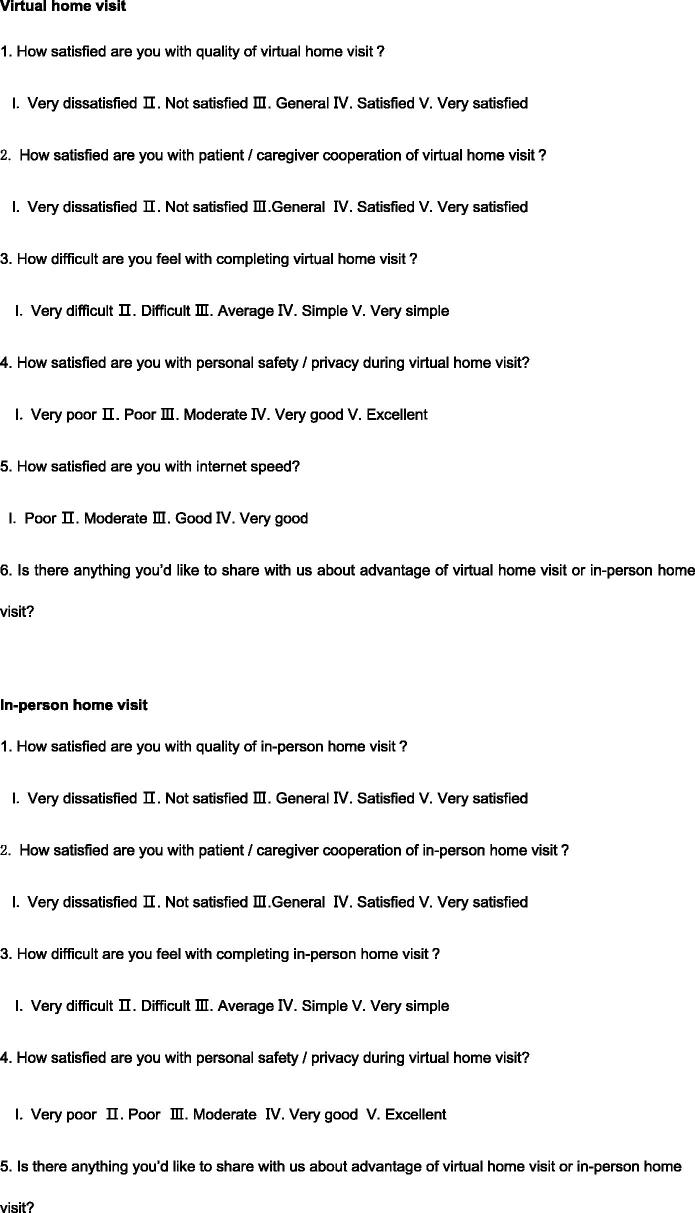
Questionnaire to measure completing of a nurse.

Evidence supports the use of checklists in medical care to improve safety and reduce risk by ensuring that all steps are taken, clinic accountability is improved, and better communication is facilitated [8,27–30]. Therefore, for this study, we developed a home visit checklist based on a literature review and our previous experience [31–35]. The checklist included three sections: (1) domestic habits: an evaluation of the home environment and personal living habits (57 items), (2) bag exchange procedure: an evaluation of peritoneal dialysis fluid replacement practice (56 items), and (3) exit site care: an evaluation of nursing practices at the exit site of the PD catheter (53 items). Except for a few items, results are presented in the form of “yes or no” answers to ensure accuracy of judgment (see Figure 1). To better manage visit time, we added a few items to the checklist about PD knowledge during the fluid input and output process. The checklist was tested and verified in simulation scenarios. The patient questionnaire included 10 questions exploring satisfaction with the quality of the visit, convenience, barriers to acceptance, and future visit preference (see Figure 2). PD patients completed the questionnaire by combining their in-person home visit experience with the virtual visit experience. The nurse questionnaire investigated the experience of visit implementation, patient cooperation, personal safety and internet speed (see Figure 3).

Figure 1.Checklist of a home visit.

**Figure:**
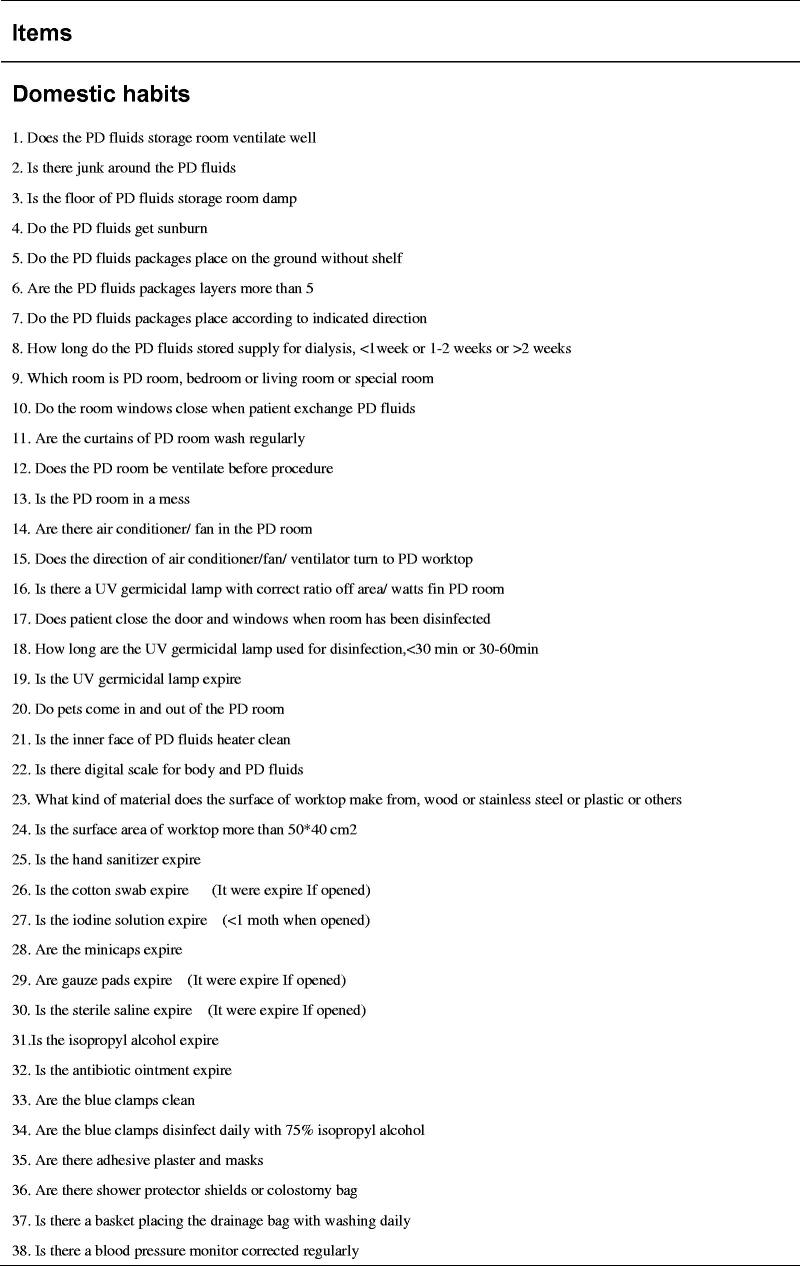

**Figure:**
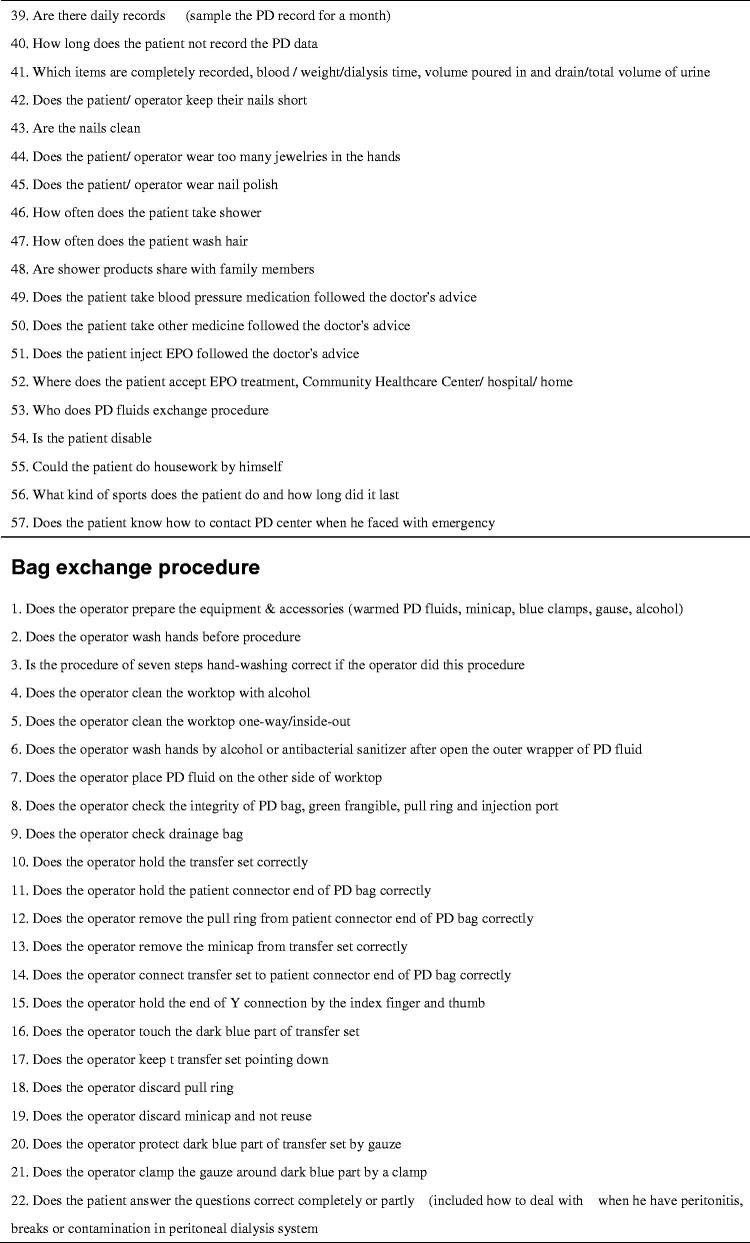

**Figure:**
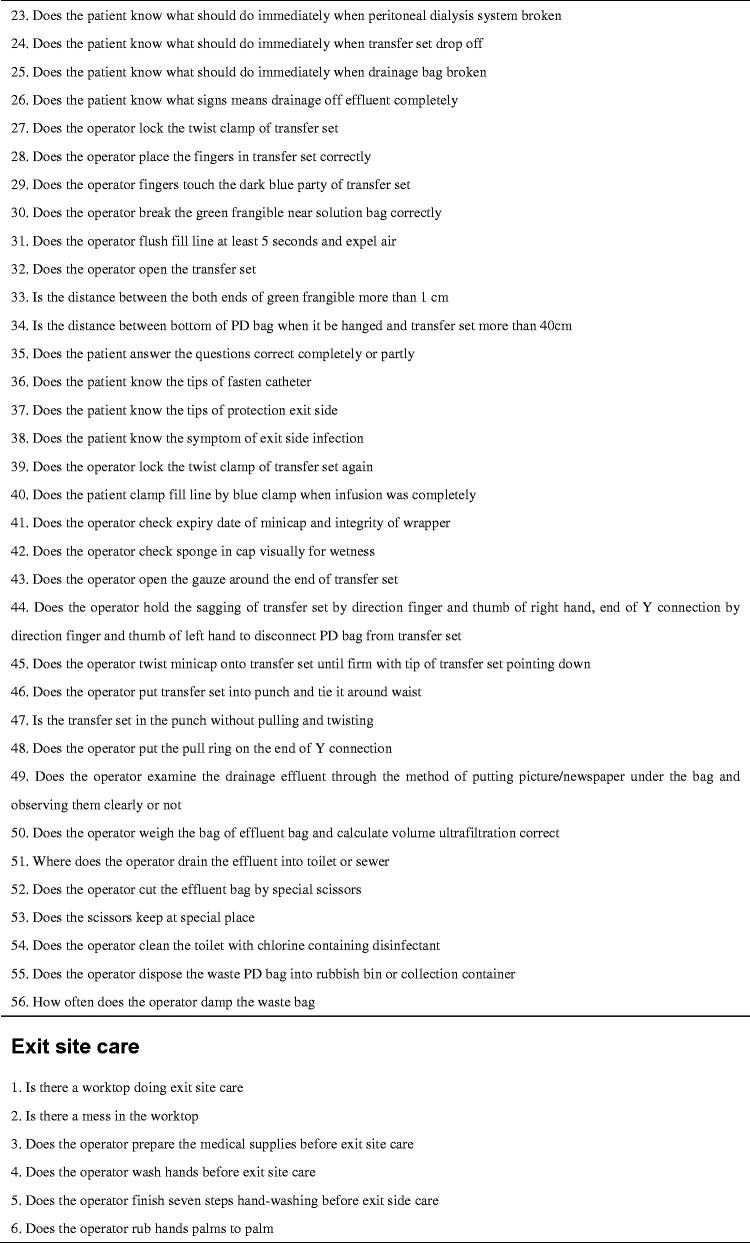

**Figure:**
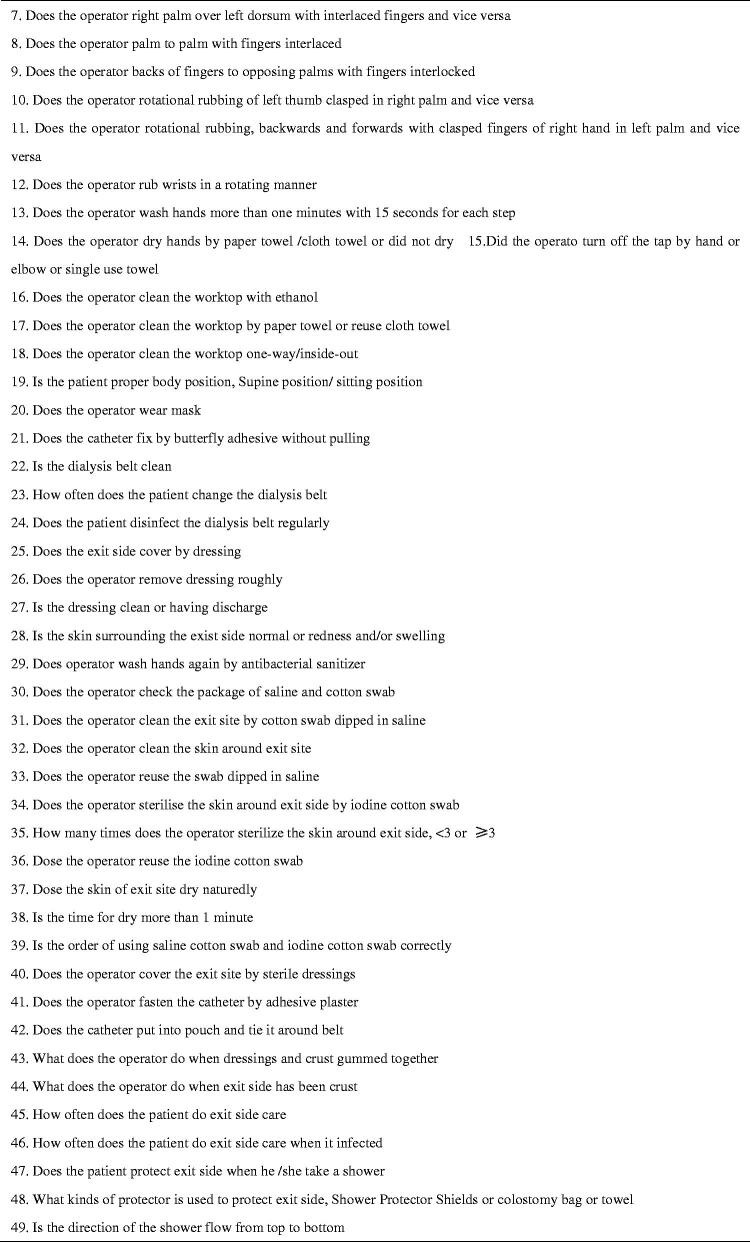

**Figure:**
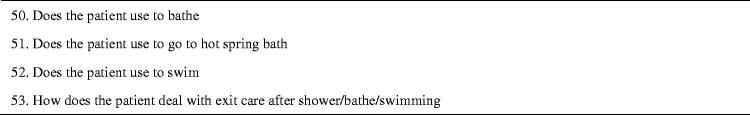

#### Training nurses and patients/caregivers for home visits

Visits were implemented by four PD nurses who were trained to follow the checklist and assess the results. Assessment agreement was ensured through scenario simulation and real-world situations between October 28 and November 3, 2020. Following enrollment, all patients/caregivers were trained in the use of the WeChat app, the adjustment of the device camera and microphone, and how to cooperate with the nurses’ visits.

#### Piloting home visits

Firstly, PD nurses made appointments and checked internet transmission conditions to visit patients. According to the research plan, in-person home visits were implemented by a nurse and a volunteer (to ensure personal safety and to help patients who lacked caregivers or other helpers to complete their virtual home visit) and virtual home visits were simultaneously completed by another nurse. For virtual home visits, the patients used the WeChat App on their smartphones to log onto the hospital website and connect to the PD center. Under the guidance of the virtual home visit nurse, the patient or a caregiver/volunteer held the smartphone to show the environment and the PD procedure during the visit. Virtual visit nurses logged onto the app by computer at the PD center and guided the camera operators to take videos according to the checklist. The in-person home visit nurse kept a note of each item on the checklist but did not participate in the inquiry or discussions with either the patient or the virtual visit nurse. At the end of each visit, patients and nurses completed their respective questionnaires. Visit data were recorded and stored in WJX.cn (a professional online questionnaire survey platform). The video recording of each patient’s virtual home visit was reserved for identification by two researchers. If any dispute arose, it was resolved through discussion or consensus. Final outcomes also emerged following discussion and consensus.

#### Statistical analysis

We described the study population using the mean and standard deviation or median and interquartile range for continuous variables, and frequency and proportion for categorical variables. Cohen’s kappa was used to measure the inter-rater agreement of the collected checklist information for both visit types. The agreement was established when data results from virtual home visits and in-person home visits were the same. Disagreement was established in cases where virtual home visit data did not reflect in-person home visit data. If items were omitted by one type of visit and recorded by other type visits, we thought they were different. The agreement was almost perfect when Cohen’s kappa 0.81–1.00, substantial when 0.61–0.80, moderate when 0.41–0.6, fair when 0.21–0.40, and slight when 0.0–0.20. The Wilcoxon signed-rank test and Chi-Square test were used to evaluate the questionnaire outcomes. Analyses were performed with Medcalc 11.4.2.0 and SPSS 26.0, and the significance level was 0.05 for the two-sided test.

## Results

### Participant demographic characteristics

Table 1 summarizes the characteristics of the enrolled patients. The mean subject age was 55.3 years with male predominance. Half of the participants had not attained high school education. Just under one quarter of participants (23.3%) lived more than 10 kilometers from the PD center, and 46.7% lived more than 20 kilometers away. Approximately 80% of participants had 4 G/5G WiFi service. Checklist information collection consistency between virtual and in-person home visits.

**Table 1.** Demographic and clinical characteristics of the study sample.

Participant characteristics	*N* = 30
Age, mean years (SD)	55 (44.8–68)
Male, %	60
Race, %	
Han	96.7
Tibetan	3.3
Dialysis vintage, mean months	47 (16.3–73.8)
Diabetes, %	20
Charlson comorbidity score	3 (2–5)
Education, %	
<High school	50
High school	23.3
College or higher	26.7
Annual income per person (¥), %	
<24,000	10
24,000–60,000	36.7
>60,000	53.3
Distance of return journey, kilometers, %	
<10	30.0
11–20	23.3
21–30	46.7
Network, %	
WIFI (4G/5G)	80
Mobile wireless (4G/5G)	20
Virtual completer, %	
Patient/volunteer	43.3
Family member	56.7
Automated peritoneal dialysis, %	3.3
Creatine, umol/L	1110.1 ± 55.1
Serum urea nitrogen, mmol/L	20.5 ± 1.1
Hemoglobin, g/L	101.0 (89.0–118.0)
Albumin, g/L	36.1 (32.9–38.7)
parathyroid hormone, pg/ml	417.0 (232.5–592.5)

A high coefficient of information collection was observed between virtual home visits and in-person home visits. The perfect agreement was found in 52/57, 49/56, and 44/53 items, substantial agreement in 4/57, 7/56, and 8/53 items for domestic habits, bag exchange procedure, and exit site care, respectively (Table 2). Action items were mainly disagreement items in the visits. Patient satisfaction and virtual home visit vs in-person home visit feasibility.

**Table 2.** Comparing disagreement items between virtual home visits and in-person home visits.

	Cases	Inconsistent items	K	CI
Domestic habits (57 items)				
	0	33	1**	
	1	11	0.91 **	(0.74, 1.00)
	2	8	0.81 **	(0.57, 1.00)
	3	3	0.73 *	(0.45, 1.00)
	4	1	0.66 *	(0.35, 0.97)
	5	1	0.56	(0.21, 0.90)
Bag exchange procedure (56 items)				
	0	30	1**	
	1	14	0.91**	(0.74, 1.00)
	2	5	0.81 **	(0.57, 1.00)
	3	3	0.73*	(0.45, 1.00)
	4	4	0.66 *	(0.35, 0.97)
Exit site care (53 items)				
	0	24	1**	
	1	10	0.91**	(0.74, 1.00)
	2	10	0.81 **	(0.57, 1.00)
	3	4	0.73 *	(0.45, 1.00)
	4	6	0.66 *	(0.35, 0.97)
	5	1	0.56	(0.21, 0.90)

Cohen's kappa: 0.0–0.20 slight, 0.21–0.40 fair, 0.41–0.6 moderate, 0.61–0.80 substantial, 0.81–1.00 almost perfect *.

Table 3 summarizes the outcome of the PD patient questionnaire. No statistically significant difference was found in satisfaction levels between virtual home visits and in-person home visits (24 vs 25, *Z* = −0.39, *p* = .70). In addition, participants noted a number of positive aspects of virtual visits, including flexibility in arranging visits, less time required for the visit, and a reduction in the work required of medical staff (see Table 4).

**Table 3.** PD patient satisfaction with virtual home visits and in-person home visits.

Survey question	Virtual visit (*N* = 30)	Home visit (*N* = 30)	Z/c2	*P* value
Comfort of visit				
Very dissatisfied	0	0	−0.39	0.7
Not satisfied	0	0		
General	1	0		
Satisfied	5	5		
Very satisfied	24	25		
Difficulty in completing visit				
Very difficult	0	0		
Difficult	1	0		
Average	0	1	−0.83	0.41
Simple	9	6		
Very simple	20	23		
What did you find uncomfortable?%				
Technical factors	43.3	33.3		
Privacy	40	30	3.07	0.22
Risks of infection and personal safety	16.7	36.7		
Patient willingness for repeat of this visit type?%				
Virtual visit				
Home visit	–	40		
Both	–	46.7		
	–	13.3		

**Table 4.** Comments from PD patients and nurses.

	Virtual home visits	In-person home visits
PD patient	Flexibility of visit arrangement	Face-to-face communication
	Less time for visit	Providing humanistic care
	Reducing work of medical staff	Convenient for the elderly
PD nurse	Guaranteeing personal safety	Better visit view
	Saving time and cost	Intuitive cognition for visits
	Providing pleasant working environment	Unaffected by internet speed

The majority of nurses reported the greater ease of completing virtual home visits, with 86.6% rating the internet speed as good or very good. In addition, no difference was reported in patients’ cooperation and personal safety between the two visit types (Table 5). Nurses noted the benefits of virtual home visits, including safety, cost-effectiveness, and a good working environment (see Table 4).

**Table 5.** Virtual home visit versus in-person home visit feasibility for PD nurses.

Survey question	Virtual visit ^a^	Home visit ^a^	Z/x^2^	*P* value
Difficulty in completing visit				
Very difficult	0	0		
Difficult	2	0		
Average	6	7	−0.99	0.33
Simple	19	17		
Very simple	3	6		
Patient cooperation			−1.65	0.10
Extremely dissatisfied	0	0		
Not satisfied	0	0		
General	0	1		
Satisfied	14	19		
Extremely satisfied	16	10		
Personal safety/Privacy			−0.54	0.18
Excellent	9	8		
Very good	12	7		
Moderate	6	3		
Poor	3	7		
Very poor	0	5		
Internet speed^b^, %				
Very good	20	–		
Good	56.6	–		
Moderate	16.7	–		
Poor	6.7	–		

^a^Total study sample (*N* = 30).

^b^Internet speed was defined by completing the visit without the connection freezing or going offline, freezing occasionally without going offline, freezing sometimes and going offline ≤1, freezing frequently and going offline ≥2.

*p < 0.05.

The average total visit time included travel, parking, waiting, appointments and training for virtual home visits was 100.6 min compared to 158.8 min for in-person home visits. The proportion of all visits spent without medical service was higher in in-person home visits than virtual home visits. In addition, the average transportation and manpower costs per visit were lower for virtual home visits than for in-person visits (229.2 vs 377.1) (Table 6).

**Table 6.** Cost-effectiveness outcomes for virtual home visits versus in-person home visits.

Outcome	Virtual home visit	In-person home visit	*t*	*P*	95% CI
Mean time spent (in mins), including travel, parking, and waiting	105.8	168.0	−8.59	*P* < 0.001	[−76.78, −47.62]
Mean time spent (in mins), including appointments and training	15.1	7.2	13.14	*P* < 0.001	[6.7, 9.2]
Proportion of visit spent without medical service	0.14	0.44	−12.46	*P* < 0.001	[−0.35, −0.25]
Mean cost in gas, tickets, wages lost (in RMB)	229.2	377.1	−8.92	*P* < 0.001	[−181.2, −114.4]

## Discussion

In this paired study of virtual home visits and in-person home visits with peritoneal dialysis patients, we found strong consistency in the information collected by the two visit types. The majority of patients reported similar satisfaction with the implementation of virtual home visits and in-person home visits. Furthermore, less time was required of PD nurses to complete virtual home visits. There was reduced manpower expenditure and eliminated personal safety concerns.

PD home visit is a form of care work. In this complex situation, mistakes can easily be made. This home visit checklist was easy to implement and qualitatively beneficial in guiding complex home visits to ensure that all facets of care were addressed and that the quality of visits was effectively evaluated. The development of a well-designed checklist usually follows three steps—literature review, incident analysis, and structure interview with operators [36]. It is important that home visit checklists are customized to local PD center protocols and procedures. Although further research is required to quantify the value of the checklist with regard to outcome needs, it has been shown to be a valid tool for effectively completing home visits and for measuring agreement between the two visit types.

Our study confirmed that information collected from virtual home visits is highly consistent with that of in-person home visits. However, inconsistency was observed in some items. Human error may have been a contributing factor to this inconsistency. Inevitably, PD nurse subjectivity leads to differences in judgment. For example, when looking back at the video, we found that the nurse made a judgment that disagreed on the same item for the same patient. In addition, unclear instructions make the camera holder fail to capture the procedure and lead to error judgment in virtual home visits. Other important factors affecting the consistency rate in collecting information included technical factors. Some virtual home visit nurses reported that internet network instability resulted in occasional video freezing or crashing, leading to the loss of some information. Poor consistency of action items was observed in virtual visits. It was a challenge for PD nurses to continuously track and recognize a series of movements during a prolonged home visit. Previous studies used motion capture systems to gauge the kinematic features of motion, providing a means to collect action information resolution [37,38]. We recommend the development of software to capture body movements using a higher resolution web camera, such as Kinect for Windows, to facilitate better information collection in future research.

Other recent studies show similar satisfaction levels for virtual visits and in-person visits [24,39,40]. The questionnaire survey revealed that technical factors were the main cause of dissatisfaction. A study found slightly less use of the internet among patients aged 65 years and older, and that health literacy, annual income, and educational attainment levels impacted interest in using telehealth applications [41]. Given the fact that most PD patients are elderly and have lower levels of education, simple e-communication platforms should be used to allow for greater ease of communication between patients and medical staff. Moreover, quick and easy access to mobile internet applications will increase patients’ comfort with virtual home visits.

Privacy was also a leading factor affecting patient satisfaction with virtual home visits. Patients were concerned about the privacy and security of their personal health information, worrying that it could potentially be compromised online and disclosed to others, or used by others to infringe on their rights [42]. This suggests that government should legislate for the improvement of telemedicine regulations before this form of consultation is widely available to patients. In addition, telehealth institutions should build communication protocols and protect the privacy and security of patient data to meet the stringent patient privacy regulations dictated by existing laws [42,43]. A successful virtual visit project must also remove barriers by establishing confidence between patients and visitors, thus ensuring greater willingness to make use of virtual visits [44].

In this research, we observed similar feasibility of virtual home visits compared to in-person home visits. However, nurses in the in-person home visit group presented an evaluation of poor or very poor for personal safety/privacy, although the difference was not statistically significant. Anxiety about violence and road safety risks usually arise when PD nurses carry out in-person home visits alone. In general, PD nurses receive less training than district nurses in risk management, personal safety, and handling aggressive behavior. Research has found that the risk of sustaining an injury from physical assault is 9% higher for lone workers in health and social care than for non-lone workers [45]. Virtual home visits could eliminate these potential risks. Previous studies have shown that virtual home visits also have socioeconomic advantages, each visit requires less time and the costs are reduced. Our results confirm these findings [24,38,46]. The average total visit time and cost for virtual home visits was less than in-person home visits. However, further research is needed to explore the relationship between the care delivery modality, total costs, and clinical outcomes.

The strengths of this study include the collection and comparison of virtual home visits and in-person home visit information through the use of a checklist to ensure the uniformity and objectivity of evaluation criteria. In addition, to better understand the factors affecting satisfaction and flexibility, we conducted a survey of patient attitudes toward the two types of visits.

However, the current study has some limitations. The study was performed at a single center, thus limiting generalizability. The contents of the checklist require further iterations to make them suitable for different PD center contexts. Although the study passed the implementation consistency evaluation, visitors were not randomized for infectious disease prevention and limited manpower due to the COVID-19 pandemic. This may have led to bias in the visit questionnaire. To ensure consistency of information sources, virtual home visits and in-person home visits were conducted simultaneously which may have confused patients’ evaluation of satisfaction, although in-person home visit evaluations were based on past and present experiences. Finally, this short-term study was designed to focus on information consistency to test the efficiency of virtual home visits. Other aspects of PD care, such as peritonitis, survival, and hospitalization rates, were not investigated. Follow-up research needs to be conducted to better understand these endpoints. For better evaluating the feasibility of telemedicine in PD patients, we also need to carry out more research to compare the visit protocol, suitable population, artificially intelligent assistant and so on between virtual visits and an in-person home visit.

During the pandemic, the various applications of telemedicine for a home visit in dialysis are explored rapidly. With limited investments, telemedicine offers many advantages such as facilitated contact with patients, humanizing care. A virtual visit is generally useful and promising among them. But telemedicine requires an adaptation of work organization and they cannot replace the welfare deficiencies of the health system at this stage.

In conclusion, this study suggested that virtual home visits have similar information collection consistency as in-person home visits in PD patients. Both patients and nurses reported satisfaction and feasibility with virtual home visits. The network should be checked before visits to ensure virtual transmission quality. If given similar quality, feasibility, and socioeconomic advantages, virtual home visits are a worthwhile application in the management of PD and are deserving of further research.

## Data Availability

The data that support the findings of this study are available from the corresponding author upon reasonable request.
